# The Values and Preferences of People Living With Motor Neurone Disease (MND): A Systematic Review Protocol

**DOI:** 10.1002/gin2.70039

**Published:** 2025-08-26

**Authors:** Timothy Hugh Barker, Grace Holland, Camille Schubert, Danielle Pollock, Cindy Stern, Nipun Shrestha, Wojtek Wiercioch, Samer Karam, Steve Vucic, Jay Beasley‐Hall, Lynne Giles, Tracy Merlin, Zachary Munn

**Affiliations:** ^1^ Health Evidence Synthesis, Recommendations and Impact (HESRI), School of Public Health, Faculty of Health and Medical Sciences The University of Adelaide Adelaide Australia; ^2^ Adelaide Health Technology Assessment (AHTA), School of Public Health, Faculty of Health and Medical Sciences The University of Adelaide Adelaide Australia; ^3^ Michael G. DeGroote Cochrane Canada & McMaster GRADE Centres McMaster University Hamilton Ontario Canada; ^4^ Department of Health Research Methods, Evidence, and Impact McMaster University Hamilton Ontario Canada; ^5^ Department of Health Research Methods, Evidence and Impact McMaster University Hamilton Ontario Canada; ^6^ Brain and Nerve Research Centre, Concord Clinical School The University of Sydney Sydney Australia; ^7^ University Library, The University of Adelaide Adelaide Australia; ^8^ School of Public Health, Faculty of Health and Medical Sciences The University of Adelaide Adelaide Australia

**Keywords:** motor neurone disease, preferences, valuation of outcomes, values

## Abstract

**Objective:**

To systematically review the values and preferences of people with lived experience of motor neurone disease (MND), including those living with MND, caregivers and genetic carriers, regarding their health‐related outcomes.

**Introduction:**

MND is a devastating neurodegenerative disease that significantly impacts those living with the disease, their caregivers, and their families. Understanding the values and preferences of those affected by MND is crucial for providing patient‐centred care and developing trustworthy guidelines.

**Eligibility Criteria:**

Studies will be included if they report on the values and preferences of adults living with MND; caregivers and families of those diagnosed with MND; clinicians as a proxy for adults living with MND; or asymptomatic genetic carriers of MND. Peer‐reviewed studies utilising either quantitative or qualitative methodologies (or both) will be eligible for inclusion in this review.

**Methods and Analysis:**

A comprehensive search of electronic databases will be conducted to identify relevant studies. Data extraction, risk of bias assessment (of quantitative studies), and assessment of methodological limitations (of qualitative studies) will be performed independently by two reviewers. Quantitative data will be pooled using meta‐analysis where appropriate, and qualitative data will be synthesised following a modified meta‐aggregative approach. The relevant GRADE approach will be used to assess the certainty of evidence.

**Systematic Review Registration Number:**

CRD420250653287.

## Summary

- This protocol outlines the methods and processes to undertake the first systematic review designed to synthesize evidence on the health‐related values and preferences of the motor neurone disease (MND) community. The review will seek to understand what health outcomes are most important to people with lived experience of the disease.
- The protocol is specific to an inclusive population of adults with MND, caregivers, and genetic carriers. It details a comprehensive plan for study selection, data extraction risk of bias assessment, assessment of the certainty of the evidence following GRADE methodology.
- The purpose of this review is to generate crucial evidence to inform the development of trustworthy guidelines, and will directly contribute to the Australian MND Guideline, ensuring recommendations reflect the priorities of those affected by the disease.

## 1 Background

Motor Neurone Disease (MND) is a progressive neurodegenerative disorder characterised by the degeneration of both upper and lower motor neurones [1]. This can lead to muscle weakness, paralysis, and eventual respiratory failure, resulting in significant disability and reduced quality of life for affected individuals [2, 3, 4]. According to the International Classification of Diseases [5], types of MND may include all of amyotrophic lateral sclerosis (ALS); progressive muscular atrophy (PMA); primary lateral sclerosis (PLS); progressive bulbar palsy (PBP); and hereditary spastic paraparesis (HSP) [5, 6]. In Australia, MND affects approximately 1500 to 2000 people at any given time, with two Australians passing away due to MND every day [4, 7, 8]. The impact of MND is disproportionately high due to its devastating effects on physical function and the absence of a cure [9]. In addition to the profound impacts on the health and emotional well‐being of patients, MND also directly impacts the psychological and social well‐being of the families and caregivers of patients [9, 10, 11].

Individuals living with MND (and their caregivers) face numerous decisions regarding their care, which need to be balanced across the connected constructs of independent health, social well‐being, and emotional safety. There is well‐documented diversity among individuals with lived experienced of MND regarding their priorities and preferences with respect to their health outcomes, with some patients opting for strategies that prolong survival, while others may opt for comfort or maintenance of their autonomy [12, 13]. The preference for a specific provision of care that a patient may opt to engage with is therefore an implicit result of the importance of health outcomes that an individual connects with that specific provision of care [13]. The GRADE (Grading of Recommendations Assessment, Development and Evaluation) Working Group operationalise patient values and preferences as “the relative importance patients (and other populations of interest) place on outcomes” [4, 5, 6].

Understanding the values and preferences of people with a lived experience of MND is not only critical to the appropriate delivery of patient‐centred care [14], but it is also critical in the development of trustworthy guidelines. Evidence towards the values and preferences of those with a lived experience of a condition directly informs the importance of outcomes, the trade‐off between benefits and harms, the direction of any recommendations made, dissemination strategies, and any decision aids and implementation tools associated with the guidelines [13]. Traditionally in guideline development, values and preferences have only been addressed through informal approaches, typically through input of patient representatives participating in guideline panels.

A preliminary search of Medline via PubMed identified that currently, there exists no systematic review that has synthesised the existing evidence regarding the values and preferences of people with lived experience of MND. This systematic review therefore aims to synthesise this existing evidence on the values and preferences of people with lived experience of MND regarding their health‐related outcomes.

## 2 Review Question

What are the values and preferences of the MND Community, regarding their health‐related outcomes associated with any intervention for MND care?

## 3 Methods

This review is part of the overall project coordinated by Health Evidence Synthesis, Recommendations, and Impact (HESRI) and the Adelaide GRADE Centre to develop the Australian MND Guideline. This review was conducted following guidance from Cochrane [15] and the GRADE Working Group [13, 16, 17] and utilises adapted methodology from previous systematic reviews investigating the values and preferences of the target population [18, 19, 20, 21, 22, 23]. This review will be reported in line with PRISMA (Preferred Reporting Items for Systematic Reviews and Meta‐Analyses) 2020 [24] and this protocol has been reported in line with PRISMA‐P (PRISMA extension for protocols) [25] (Appendix Table A1). This review has been registered within PROSPERO, ID: CRD420250653287.

## 4 Eligibility Criteria

### 4.1 Participants

Studies will be eligible for this review where they have reported data from any of the following sub‐populations of people:

1. Adults (18+) diagnosed with any type of MND, such as amyotrophic lateral sclerosis (ALS), progressive bulbar palsy (PBP), progressive muscular atrophy (PMA), primary lateral sclerosis (PLS), hereditary spastic paraparesis (HSP), MND with frontal temporal dementia (MND/FTD), Hirayama disease/monomelic amyotrophy (MMA) and adult onset (type VI) spinal muscular atrophy (SMA). Regarding SMA, studies will only be eligible for inclusion where the data is specific to adult‐onset SMA (e.g., type IV).
2. Individuals with lived experience of MND, including caregivers and family members directly involved in care decisions. There will be no restrictions placed on the mode of caregiving (e.g., live‐in caregiver, full time caregiver, paid vs. unpaid etc).
3. Asymptomatic carriers (also known as genetic carriers) of MND. Defined as individuals that carry a mutation in a gene (C9orf72, SOD1, FUS, and TARDBP genes) associated with MND, but do not exhibit symptoms of the disease themselves.

Studies will be ineligible for this review where they focus exclusively on other neurological conditions unrelated to MND, however should studies investigate both MND and other unrelated neurological conditions, attempts will be made to extract only the data related to MND. Studies will also be ineligible for this review where they involve only populations without direct lived experience of MND (e.g., general public, or healthcare professionals without firsthand interaction).

Studies that have investigated outcome valuations by clinicians as a proxy to the outcome values of a person diagnosed with MND are eligible for inclusion. Studies that investigate outcome valuations by clinicians, in relation to clinician‐specific outcomes (e.g., burnout) will be excluded.

### 4.2 Phenomena of Interest

Studies will be eligible for this review, where they have explored the values and/or preferences of the eligible population regarding their satisfaction or health and quality of life‐related outcomes. These need to include specific valuations of the eligible population regarding their satisfaction or health and quality of life‐related outcomes. This may be provided as a baseline measurement and associated with specific health states or associated with any intervention for MND care. This will include (but is not limited to) preferences related to symptom management, end‐of‐life care decisions, treatment trade‐offs, or other health or healthcare‐related experiences of living with MND.

### 4.3 Context

There will be no limitations regarding study eligibility for this review based on context. Studies will be eligible for this review that have been conducted in any country and in any healthcare setting (e.g., hospitals, outpatient clinics, home care etc).

There will be no exclusions based on language and no date limitations. Dependent on the language identified, the authors may identify contacts that may be able to assist with translation. Otherwise, DeepL Translator (DeepL, Cologne, Germany) will be used to translate any titles and abstracts in languages authors are not fluent in. Where reports of studies are deemed relevant for review at the full text stage, they will be translated using DeepL and, if considered relevant, be translated by a professional translator. We will reach out to experts from the Australian MND guideline for suggestions of references to include where appropriate.

### 4.4 Outcomes

An exhaustive list of outcomes cannot be pre‐determined due to the expected diversity of the available data. However, studies will be eligible for this review where they report data on health outcomes such as (but not limited to) quality of life, satisfaction, survival, autonomy, and so forth.

Studies will be ineligible for this review when the reported outcomes focus only on clinical measures or biomarkers without direct connections to patient (and/or caregiver) values or preferences (e.g., evaluation of fine motor skills, speech or swallowing function without connection to the patient (and/or caregiver) values or preferences).

### 4.5 Study Design

To improve reliability and scientific rigour of the review, studies will be eligible for this review only if they are peer‐reviewed research papers that follow an established and appropriate methodology (described below). Eligible studies will include any that have reported eligible populations' preferences and/or values regarding their MND‐related health outcomes in association with a specific intervention associated with MND care. Studies with the following characteristics are eligible where reporting the relative importance of the health‐related outcomes [13]:

1. Patient‐reported measures (PRMs) and quality of life studies
  Studies that quantitatively measure the lived experience of the population(s) of interest, in one or multiple domains that are directly relevant to patient satisfaction (with their health‐related quality of life or wellbeing); typically referred to as patient‐reported experience measures (PREMs) or patient‐reported outcome measures (PROMs). Quality of life measuring tools may be disease/topic specific questionnaires (such as the ALSAQ‐40 for MND [26]), or generic multi‐attribute utility instruments (MAUIs) such as the EQ‐5D [27], Short Form‐36 Health Survey (SF‐36) [28], Health Utilities Index (HUI) [29] or Quality of Wellbeing (QWB) [30]. Where quality of life data have been captured using a validated tool and subsequently translated to a utility estimate (described below) using an external mapping or preference algorithm, both the raw and translated measures may be relevant.
2. Direct utility and health state value studies
  Studies that directly measure how the population of interest perceive the value or quality of different health states by use of assigned numerical values (utility values) that reflect the desirability of living in those states [31]. Utility values are typically reported on a scale where 1 represents perfect health, and 0 represent a state equivalent to death [31]. In some instances, negative values can be used to indicate a state that is considered worse than death. Study methodologies that elicit direct measurement or valuation include survey techniques such as the Standard Gamble (SG), Time Trade‐Off (TTO) or Visual Analogue Scale (VAS). Although representing the same concept and nominally the same, utility values estimated by different methods of direct elicitation are poorly comparable to one another and to the indirect estimation of utility captured through MAUIs.
3. Direct choice studies
  Studies that quantitatively examine the population of interest's choice, after presenting them with a description of hypothetical states or during decision making for their own actual health states [20]. Studies may utilise a willingness to pay approach and may also utilise a direct/forced choice exercise where the population of interest is prompted to make a choice from a set of options or rank a set of alternatives to enable best‐worst scaling [32].
4. Other studies utilising quantitative methodology to assess outcome importance
  Studies that may employ any other quantitative methodology (e.g., bespoke questionnaires or other Nonutility measurement techniques) whereby the patients' values and preferences regarding their health‐related outcomes are examined.
5. Qualitative studies on outcome importance
  Qualitative research studies that have explored the population of interest's values and preferences related to different health‐related outcomes. However, qualitative research that explores the population of interest's values and preferences related to treatment options/modalities or provision of care were not eligible for inclusion.

Studies will also be eligible for inclusion where they have employed a range of eligible methodologies, (e.g., mixed‐methods studies that include both quantitative and qualitative data collection methods would be eligible, if the methodologies followed are consistent with those listed above).

## 5 Search Strategy

The search strategy aims to locate all available studies that meet the eligibility criteria above. The search strategies for each relevant source (detailed below) were developed by a research liaison librarian (JBH) and were peer‐reviewed using the Peer Review of Electronic Search Strategies Guideline Statement [33].

An initial limited search of PubMed via NCBI was undertaken to identify relevant articles on this topic. The terminology contained in the titles and abstracts of relevant articles, including related subject headings, were used to develop a full search strategy for Medline (PubMed via Ovid). The search strategy, including all identified keywords and subject headings, was adapted for each included database and/or information source. The reference list and citations of all studies undergoing extraction will be screened for additional studies using citationchaser [34] and/or a related citations search [35]. The full search strategy for major databases is available (Appendix Table A2: Search strategy).

The databases to be searched include the Cochrane Central Register of Controlled Trials (CENTRAL); MEDLINE (PubMed via Ovid); EMBASE (Ovid); ClinicalTrials.gov (www.ClinicalTrials.gov/) and The World Health Organization's (WHO's) International Clinical Trials Registry Platform (WHO ICTRP) (www.who.int.ictrp).

## 6 Study Selection and Screening

After conducting the search, all identified records were compiled and uploaded into EndNote (ver. 21.2, Clarivate, Philadelphia, Pennsylvania). The records were then imported into Covidence (Veritas Health Innovation, Melbourne, Victoria), where duplicate records were identified and removed. All records will then be screened on their titles and abstracts by two or more independent reviewers against the eligibility criteria for the review. Piloting of this screening process will take place on 5% (or minimum five records) of the records imported into Covidence. Piloting of screening will take place between all reviewers. Results of the piloting will then be compared; more piloting will occur if agreement reached is less than 70% between all reviewers. Following piloting, the remaining records will be screened at the title and abstract level independently, so that each record will be screened by at least two people. A record without an abstract and records that are potentially relevant studies will be retrieved in full will be included for full‐text retrieval.

The full‐text reports of each relevant record, potentially relevant record or record with no abstract will be retrieved. These reports will then be assessed against the eligibility criteria by two or more independent reviewers. Another pilot test will occur on 5% (or minimum five reports) of the reports included for screening at the full‐text level. Additional piloting will occur if agreement reached is less than 70% between all reviewers. Following piloting, the remaining reports will be screened in detail against the inclusion criteria by two or more independent reviewers, so that each report is screened by at least two people. For reports that do not meet the eligibility criteria after being screened at the full‐text level, the reasons for their exclusion will be documented and reported in the appendices of the final report. Any disagreements that arise between the reviewers at any stage of the selection process will be resolved through discussion, or with additional reviewers. The results of the search and screening process will be reported in full in the final review and presented in a PRISMA 2020 flow diagram [36]. Where relevant systematic reviews are identified in the search, we will review the list of included and excluded studies for consideration, however the systematic reviews themselves will not be included. Where members of the author team are named as authors on primary studies identified through the search, they will be excluded from any decision‐making regarding the inclusion of the study or the assessment of the study risk of bias/methodological limitations.

## 7 Assessment of the Risk of Bias and Methodological Limitations

Two review authors will independently assess the risk of bias for each study using the Risk of Bias in Studies of Values and Utilities (ROBVALU) tool [37], for quantitative studies. Assessment of methodological limitations will be independently assessed using the Cochrane Qualitative Methodological Limitations Tool (CAMELOT), for qualitative studies [38]. For any studies identified that have been informed by both quantitative and qualitative methodology, both tools will be applied where appropriate.

ROBVALU considers four subdomains: selection of participants, completeness of data, measurement of instrument and data analysis. Across these subdomains, seven key signalling questions are presented, the answers to these being yes, probably yes, probably no and no. Each study is then ascribed an overall risk of bias (no serious risk of bias, serious risk of bias, very serious risk of bias or extremely serious risk of bias). The application of the ROBVALU tool to assess risk of bias in studies evaluating the value people place on outcomes follows seven steps [37]:

1. Specify the review question
2. Specify the outcome being assessed
3. Identify the sampling frame, the measurement instrument used, and the data analysis plan
4. Answer the signalling questions of the four subdomains (where possible, this will occur at the outcome and result level)
5. Make a judgment if the four subdomains have important risk of bias concerns
6. Formulate a judgement regarding bias for the four subdomains
7. Formulate an overall risk of bias judgment for the study outcome being assessed

CAMELOT considers Meta domains (research aim and questions, stakeholders, researchers and context) and Method domains (research strategy, ethical considerations, equity, diversity and inclusion considerations, theory, participant recruitment and selection, data collection, analysis and interpretation, presentation of findings). The application of CAMELOT follows five steps [38]:

1. Extract/code data from the primary study to the domains
2. Note any comments regarding each domain (e.g., problems or missing information)
3. Describe concerns regarding, and make assessment of, fit between domains
4. Describe level of concern regarding methodological limitations
5. Combine assessment across studies

The assessment of risk of bias and methodological limitations will be piloted on two studies per design (e.g., two quantitative studies and two qualitative studies), and all assessments will be conducted by two or more independent reviewers. Any disagreements that arise between the reviewers will be resolved through discussion, or with an additional reviewer/s. If appropriate, authors of papers will be contacted to request missing or additional data where required. Results of risk of bias assessment and methodological limitations will be presented narratively in the final report and will be supported by figures and tables. All studies, regardless of their assessment of risk of bias and/or methodological limitations will be included in the review.

## 8 Data Extraction

Data from all included studies will be extracted independently and in duplicate, using structured extraction forms relevant to type of data (Appendix Table A3: Quantitative data extraction instrument and Appendix Table A4: Qualitative data extraction instrument). Both instruments have been adapted from a previous patient values and preferences systematic review [18]. Both data extraction forms will be piloted on two studies included in the review following study selection (as such, the final extraction tool may appear differently to the tools presented in this protocol). Data extraction will be supported where appropriate by a dictionary of terms to ensure uniformity of extraction items between members of the review team (Appendix Table A5: Data dictionary). Disagreements and inconsistencies in extraction will be resolved through discussion and consensus. If required, a third member of the review team will be consulted.

## 9 Data Synthesis

### 9.1 Quantitative Data

Where more than one study has described the same health state, using the same instrument, for the same pre‐determined population subgroups (e.g., people living with MND, caregivers or carriers), and (if translation was required) has translated their raw data using a common preference set, the data will be pooled using meta‐analysis as per the methods of Saeed 2020 [23]. For data that have been derived from studies that have provided an outcome valuation from clinicians as a proxy to outcome valuations from a person diagnosed with MND, these studies will be combined with the relevant studies from those with people living with MND, but will be sub‐grouped to explore differences between these two sub‐populations. Where data has been provided from a study participant that may belong to multiple ‘participant groups’ (e.g., a genetic carrier who is also a caregiver of a person with MND), a subgroup analysis will also be performed. Where the data allows, this will also be explored through meta‐regression where common study characteristics exist between studies. If three or fewer studies are to be pooled in meta‐analysis, an inverse‐variance, fixed‐effect model will be used. When four or more studies are to be pooled in meta‐analysis, a DerSimonian‐Laird random effects model will be used. Additionally, where a random effects model was appropriate, a fixed effect meta‐analysis will also be conducted to assess the sensitivity of the results to the modelling assumptions. All meta‐analyses will be conducted in R (ver. 4.4.1), using the *metafor* package [39].

As per the methods of Saeed 2020 [23], relative heterogeneity across studies will be assessed using the *I* ^2^ statistic. Interpretation of the *I* ^2^ statistic will be accordance with the guidance in the Cochrane Handbook for Systematic Reviews of Interventions [15] and will occur as follows:

0% to 40%: heterogeneity might not be important;

30% to 60%: may represent moderate heterogeneity;

50% to 90%: may represent substantial heterogeneity; or

75% to 100%: considerable heterogeneity.

The magnitude and direction of effects and the strength of evidence on the basis of the confidence interval associated with *I* ^2^ will also be considered in its interpretation. Similarly absolute heterogeneity will be investigated by visual inspection of forest plot and estimating prediction interval for effect estimates.

Where health states have been reported across multiple studies, however different instruments have been used to preclude meta‐analysis, then the ranges of each utility score from these studies will be reported.

Where a single study has provided a utility score for a health state, an appropriate reported measure of central tendency and associated measure of variability will be reported. A narrative description for each health outcome will also be reported as per the methods of Etxeandia‐Ikobaltzeta 2020 [18].

Nonutility quantitative outcome data (e.g., data from direct choice studies or other quantitative studies on outcome importance) will be narratively reported according to pre‐determined population subgroups (Patients, Caregivers or Carriers).

We will contact study authors to obtain missing data, however we will not impute any missing data from available data.

### 9.2 Qualitative Data

For studies using qualitative methods, data will be synthesised by following a modified (in the sense that we won't be developing synthesised findings that represent a line of action) meta‐aggregative approach [40]. Findings are defined as the analytic interpretation by author(s) of their data and will be in the form of a statement representing a preference or value regarding a health state or intervention, accompanied where possible by an illustration, in the form of a quote from a participant or the authors' justification for their analytic interpretation. The credibility of the illustration to support the authors' finding will be assessed following guidance from the JBI manual for Evidence Synthesis [41], and a level of credibility will be assigned. Credibility can be assigned as unequivocal, credible or unsupported.

Qualitative research findings will be extracted verbatim either at the theme or subtheme level. This will be determined by the level of abstraction of the primary study. For example, we expect most of the studies to be qualitative descriptive in nature, with more descriptive categories rather than highly abstracted/conceptual/interpretive themes. For these qualitative descriptive papers, we plan to extract their final categories (or themes). However, for the papers that are more theoretically driven and aligned to classical qualitative inquiry methodologies (such as phenomenology or grounded theory), we plan to extract at the subtheme or second order analyses/findings level as these are likely to be more closely aligned with the level of abstraction of the findings from the qualitative descriptive studies.

We will then synthesise the findings across studies to generate a set of statements that represent that aggregation, through assembling the findings and categorising these findings on the basis of similarity in meaning.

### 9.3 Patient and Public Involvement

This protocol for a systematic review is being conducted for the purposes of informing a guideline. Guideline development panel members, including people with lived experience of MND, which include many diverse stakeholders including the public/patients, other potential end‐users of the guideline, experts, and decision makers are authors of this protocol and subsequent systematic review, and have shaped the review questions and focus and will guide the interpretation of the results.

## 10 GRADE

For outcome data that are derived from quantitative data, the GRADE approach for grading the certainty of evidence in the importance of outcomes or values and/or preferences will be followed [16, 17]. For outcome data that are derived from qualitative studies, the GRADE approach for assessing the Confidence in Evidence from Reviewers of Qualitative research (GRADE CERQual) [42] will be followed. GRADE Evidence Profiles will be created using GRADEpro GDT. Each evidence profile will present the following information where appropriate: study design, measurement instrument (or methodology where appropriate), number of participants, estimate of outcome importance (for quantitative data), summary of review finding (for qualitative data). Additionally, an overall certainty assessment will be provided, which will be informed through individual assessments of the domains of risk of bias, inconsistency, indirectness, imprecision, and publication bias of the overall body of evidence for each outcome. The overall certainty of evidence will start as high and be downgraded according to the domains listed above.

## Author Contributions

**Timothy Hugh Barker:** writing original draft, methodology, conceptualisation, funding acquisition. **Danielle Pollock:** writing – review and editing, funding acquisition. **Cindy Stern:** writing – review and editing. **Camille Schubert:** writing – review and editing, funding acquisition. **Nipun Shrestha:** writing – review and editing. **Grace Holland:** writing – review and editing, funding acquisition. **Lynne Giles:** writing – review and editing, funding acquisition. **Jay Beasley‐Hall:** validation, data curation, funding acquisition. **Tracy Merlin:** writing – review and editing, funding acquisition. **Wojtek Wiercioch:** validation, methodology. **Samer Karam:** validation, methodology. **Zachary Munn:** writing original draft, methodology, conceptualisation, funding acquisition.

## Ethics Statement

As this systematic review is a review of primary studies it does not require ethics approval. No individual level participant data will be collected. All study files (data extraction forms, risk of bias assessments, RevMan files etc) will be made available via a publicly available project space using open science platforms.

## Conflicts of Interest

Cindy Stern, Danielle Pollock, Grace Holland and Nipun Shrestha are members of the Adelaide GRADE Centre. Samer Karam is a member of the McMaster GRADE Centre. Timothy Hugh Barker is Deputy Director of the Adelaide GRADE Centre. Wojtek Wiercioch is Director of the McMaster GRADE Centre. Zachary Munn is Director of the Adelaide GRADE Centre.

## Data Availability

All data for this protocol, and for the entire Austrlaian MND Guideline is available on the Open Science Framework (OSF) at https://osf.io/c2ezm/.

## Appendix I

Table A1

**Table A1:** PRISMA‐P (Preferred Reporting Items for Systematic review and Meta‐Analysis Protocols) 2015 checklist: recommended items to address in a systematic review protocol*.

Section and topic	Item no	Checklist item	Location in report
Administrative Information			
Title:			
Identification	1a	Identify the report as a protocol of a systematic review	Page 1
Update	1b	If the protocol is for an update of a previous systematic review, identify as such	NA
Registration	2	If registered, provide the name of the registry (such as PROSPERO) and registration number	
Authors:			
Contact	3a	Provide name, institutional affiliation, e‐mail address of all protocol authors; provide physical mailing address of corresponding author	Page 1
Contributions	3b	Describe contributions of protocol authors and identify the guarantor of the review	Page 10
Amendments	4	If the protocol represents an amendment of a previously completed or published protocol, identify as such and list changes; otherwise, state plan for documenting important protocol amendments	NA
Support:			
Sources	5a	Indicate sources of financial or other support for the review	Page 1
Sponsor	5b	Provide name for the review funder and/or sponsor	Page 1
Role of sponsor or funder	5c	Describe roles of funder(s), sponsor(s), and/or institution(s), if any, in developing the protocol	Page 1
Introduction			
Rationale	6	Describe the rationale for the review in the context of what is already known	Page 3
Objectives	7	Provide an explicit statement of the question(s) the review will address with reference to participants, interventions, comparators, and outcomes (PICO)	Page 3–6
Methods			
Eligibility criteria	8	Specify the study characteristics (such as PICO, study design, setting, time frame) and report characteristics (such as years considered, language, publication status) to be used as criteria for eligibility for the review	Page 4–6
Information sources	9	Describe all intended information sources (such as electronic databases, contact with study authors, trial registers or other grey literature sources) with planned dates of coverage	Page 6–7
Search strategy	10	Present draft of search strategy to be used for at least one electronic database, including planned limits, such that it could be repeated	Page 18‐23
Study records:			
Data management	11a	Describe the mechanism(s) that will be used to manage records and data throughout the review	Page 6–7
Selection process	11b	State the process that will be used for selecting studies (such as two independent reviewers) through each phase of the review (i.e., screening, eligibility, and inclusion in meta‐analysis)	Page 6–7
Data collection process	11c	Describe planned method of extracting data from reports (such as piloting forms, done independently, in duplicate), any processes for obtaining and confirming data from investigators	Page 8
Data items	12	List and define all variables for which data will be sought (such as PICO items, funding sources), any pre‐planned data assumptions and simplifications	Page 8–9
Outcomes and prioritization	13	List and define all outcomes for which data will be sought, including prioritisation of main and additional outcomes, with rationale	Page 5
Risk of bias in individual studies	14	Describe anticipated methods for assessing risk of bias of individual studies, including whether this will be done at the outcome or study level, or both; state how this information will be used in data synthesis	Page 7–8
Data synthesis	15a	Describe criteria under which study data will be quantitatively synthesised	Page 8–9
	15b	If data are appropriate for quantitative synthesis, describe planned summary measures, methods of handling data and methods of combining data from studies, including any planned exploration of consistency (such as I^2^, Kendall's τ)	Page 8–9
	15c	Describe any proposed additional analyses (such as sensitivity or subgroup analyses, meta‐regression)	NA
	15d	If quantitative synthesis is not appropriate, describe the type of summary planned	Page 9
Meta‐bias(es)	16	Specify any planned assessment of meta‐bias(es) (such as publication bias across studies, selective reporting within studies)	NA
Confidence in cumulative evidence	17	Describe how the strength of the body of evidence will be assessed (such as GRADE)	Page 9–10

*Source:* Shamseer L, Moher D, Clarke M, Ghersi D, Liberati A, Petticrew M, Shekelle P, Stewart L, PRISMA‐P Group. Preferred reporting items for systematic review and meta‐analysis protocols (PRISMA‐P) 2015: elaboration and explanation. BMJ. 2015 Jan 2;349 (jan02 1):g7647.

## Appendix II

Table A2

**Table A2:** Search strategy.

Medline (Ovid) (26/02/2025)		
1 ‐ MND	Exp motor neuron disease OR Spastic Paraplegia, hereditary.sh OR (Gehrig* disease OR motor neuron* disease* OR progressive bulbar pals* OR progressive muscular atroph* OR spinal muscular atroph* OR hereditary spastic parapares* OR “spinal and bulbar muscular atroph*” OR Kennedy* disease* OR Kennedy* syndrome* OR bulbo‐spinal atroph* or flail arm ALS OR flail leg ALS or flail arm syndrome OR anterior horn cell disease* OR charcot disease* OR guam disease* OR bulbar pals* OR fazio‐londe* disease* OR fazio‐londe* syndrome* OR spinal amyotroph* OR spinal muscular atroph* OR bulbospinal neuronopath* OR hereditary motor neuronopath* OR infantile muscular atroph* OR juvenile muscular atroph* OR infantile acute form SMA OR werdnig hoffman* disease* OR motoneuron disease* OR motor system disease* OR ALS Dementia OR chinese paralytic syndrome* OR Aran Duchenne OR Duchennen Aran OR hereditary spastic paraplegia* OR hereditary autosomal dominant spastic paraplegia* OR hereditary autosomal recessive spastic paraplegia* OR “hereditary motor and sensory neuropath*” OR hereditary x linked recessive spastic paraplegia* OR hypertrophic motor sensory neuropathy spastic paraplegia* OR spastic paraplegia type 2 OR Charcot Marie OR Charcot Hoffman OR hereditary motor sensory neuropath* OR Dejerine sottas OR familial spastic para* OR “hereditary motor and sensory neuropath*” OR hereditary sensorimotor neuropath* OR “hereditary sensory and motor neuropath*” OR neurogenic amyotroph* OR neurogenic atroph* OR neurogenic muscle atroph* OR neurogenic muscle syndrome* OR peroneal atroph* OR perone* muscle atroph* OR perone* muscular atroph* OR spastic hereditary paraglegia OR Struemell* disease* OR Struempell Lorrain OR Strumpel* disease* OR spinal amyotroph* OR bulbospinal neuronopath* OR distal spinal muscular atroph* OR hereditary motor neuronaopath* OR myelopathic muscular atroph* OR Spinobulbar muscular atroph* OR bulbo‐spinal atroph* OR MND).ti,ab,kw OR (lateral scleros*).ti,ab,kf	63441
2 ‐ Values & Preferences	Patient preference.sh OR exp attitude to health OR attitude to death.sh OR exp attitude of health personnel OR ((exp patients OR caregivers.sh OR exp family) AND exp decision making) OR ((Patient* OR famil* OR carer* OR caregiver* OR care‐giver* OR caretaker* OR care‐taker* OR clinician* OR spouse* OR parent* OR carrier* OR doctor* OR specialist* OR health‐care worker* OR healthcare worker* OR health‐care professional* OR healthcare professional* OR provider* OR plwMND OR pwMND OR “people living with”) adj6 (values OR valued OR prefer* OR desir* OR priorit* OR expectation* OR perspective* OR perceive* OR percept* OR view* OR attitude* OR accept* OR knowledge OR decision* or decid* OR choice* OR satisf* OR sentiment* OR motiv* OR approach*)).ti,ab,kw OR (patient‐reported experience measure* OR patient‐reported outcome measure* OR ALSAQ‐40 OR EQ‐5D OR Short Form Health Survey OR SF‐36 OR Health Utilities Index OR HUI OR “Quality of Wellbeing” OR QWB).ti,ab,kw	1433848
	1 AND 2	2671

Embase (Ovid) (26/02/2025)		
1 ‐ MND	Exp motor neuron disease OR exp hereditary motor sensory neuropathy OR (Gehrig* disease OR motor neuron* disease* OR progressive bulbar pals* OR progressive muscular atroph* OR spinal muscular atroph* OR hereditary spastic parapares* OR “spinal and bulbar muscular atroph*” OR Kennedy* disease* OR Kennedy* syndrome* OR bulbo‐spinal atroph* or flail arm ALS OR flail leg ALS or flail arm syndrome OR anterior horn cell disease* OR charcot disease* OR guam disease* OR bulbar pals* OR fazio‐londe* disease* OR fazio‐londe* syndrome* OR spinal amyotroph* OR spinal muscular atroph* OR bulbospinal neuronopath* OR hereditary motor neuronopath* OR infantile muscular atroph* OR juvenile muscular atroph* OR infantile acute form SMA OR werdnig hoffman* disease* OR motoneuron disease* OR motor system disease* OR ALS Dementia OR chinese paralytic syndrome* OR Aran Duchenne OR Duchennen Aran OR hereditary spastic paraplegia* OR hereditary autosomal dominant spastic paraplegia* OR hereditary autosomal recessive spastic paraplegia* OR “hereditary motor and sensory neuropath*” OR hereditary x linked recessive spastic paraplegia* OR hypertrophic motor sensory neuropathy spastic paraplegia* OR spastic paraplegia type 2 OR Charcot Marie OR Charcot Hoffman OR hereditary motor sensory neuropath* OR Dejerine sottas OR familial spastic para* OR “hereditary motor and sensory neuropath*” OR hereditary sensorimotor neuropath* OR “hereditary sensory and motor neuropath*” OR neurogenic amyotroph* OR neurogenic atroph* OR neurogenic muscle atroph* OR neurogenic muscle syndrome* OR peroneal atroph* OR perone* muscle atroph* OR perone* muscular atroph* OR spastic hereditary paraglegia OR Struemell* disease* OR Struempell Lorrain OR Strumpel* disease* OR spinal amyotroph* OR bulbospinal neuronopath* OR distal spinal muscular atroph* OR hereditary motor neuronaopath* OR myelopathic muscular atroph* OR Spinobulbar muscular atroph* OR bulbo‐spinal atroph* OR MND).ti,ab,kw OR (lateral scleros*).ti,ab,kf	100420
2 ‐ Values & Preferences	Exp patient attitude OR attitude to death.sh OR (decision making.sh AND (exp patient OR exp carer OR exp family)) OR ((Patient* OR famil* OR carer* OR caregiver* OR care‐giver* OR caretaker* OR care‐taker* OR clinician* OR spouse* OR parent* OR carrier* OR doctor* OR specialist* OR health‐care worker* OR healthcare worker* OR health‐care professional* OR healthcare professional* OR provider* OR plwMND OR pwMND OR “people living with”) adj4 (values OR valued OR prefer* OR desir* OR priorit* OR expectation* OR perspective* OR perceive* OR percept* OR view* OR attitude* OR accept* OR knowledge OR decision* or decid* OR choice* OR satisf* OR sentiment* OR motiv* OR approach*)).ti,ab,kw OR (patient‐reported experience measure* OR PREM OR patient‐reported outcome measure* OR ALSAQ‐40 OR EQ‐5D OR Short Form Health Survey OR SF‐36 OR Health Utilities Index OR HUI OR “Quality of Wellbeing” OR QWB).ti,ab,kw	1484217
	1 AND 2	4110

Cochrane Library (CENTRAL) (26/02/2025)		
1 ‐ MND	[mh “motor neuron disease”] OR [mh “Spastic Paraplegia, hereditary”] OR ((Gehrig* NEXT disease) OR “motor neuron disease” OR “motor neurone disease” OR (lateral NEXT scleros*) OR (“progressive bulbar” NEXT pals*) OR (“progressive muscular” NEXT atroph*) OR (“spinal muscular” NEXT atroph*) OR (“hereditary spastic” NEXT parapares*) OR (“spinal and bulbar muscular” NEXT atroph*) OR (Kennedy* NEXT disease*) OR (Kennedy* NEXT syndrome*) OR “bulbo‐spinal atrophy” OR“flail arm ALS” OR “flail leg ALS” or “flail arm syndrome” OR (“anterior horn cell” NEXT disease*) OR (charcot NEXT disease*) OR (guam NEXT disease*) OR (bulbar NEXT pals*) OR (“fazio‐londe” NEXT disease*) OR (“fazio‐londe” NEXT syndrome*) OR (spinal NEXT amyotroph*) OR (“spinal muscular” NEXT atroph*) OR (bulbospinal NEXT neuronopath*) OR (“hereditary motor” NEXT neuronopath*) OR (“infantile muscular” NEXT atroph*) OR (“juvenile muscular” NEXT atroph*) OR “infantile acute form SMA” OR (werdnig NEXT hoffman* NEXT disease*) OR (motoneuron NEXT disease*) OR (“motor system” NEXT disease*) OR “ALS Dementia” OR (“chinese paralytic” NEXT syndrome*) OR “Aran Duchenne” OR “Duchennen Aran” OR (“hereditary spastic” NEXT paraplegia*) OR (“hereditary autosomal dominant spastic” NEXT paraplegia*) OR (“hereditary autosomal recessive spastic” NEXT paraplegia*) OR (“hereditary motor and sensory” NEXT neuropath*) OR (“hereditary x linked recessive spastic” NEXT paraplegia*) OR (“hypertrophic motor sensory neuropathy spastic” NEXT paraplegia*) OR “spastic paraplegia type 2” OR “Charcot Marie” OR “Charcot Hoffman” OR (“hereditary motor sensory” NEXT neuropath*) OR “Dejerine sottas” OR (“familial spastic” NEXT para*) OR (“hereditary motor and sensory” NEXT neuropath*) OR (“hereditary sensorimotor” NEXT neuropath*) OR (“hereditary sensory and motor” NEXT neuropath*) OR (neurogenic NEXT amyotroph*) OR (neurogenic NEXT atroph*) OR (“neurogenic muscle” NEXT atroph*) OR (“neurogenic muscle” NEXT syndrome*) OR (peroneal NEXT atroph*) OR (perone* NEXT muscle NEXT atroph*) OR (perone* NEXT muscular NEXT atroph*) OR “spastic hereditary paraglegia” OR (Struemell* NEXT disease*) OR “Struempell Lorrain” OR (Strumpel* NEXT disease*) OR (spinal NEXT amyotroph*) OR (bulbospinal NEXT neuronopath*) OR (“distal spinal muscular” NEXT atroph*) OR (“hereditary motor” NEXT neuronaopath*) OR (“myelopathic muscular” NEXT atroph*) OR (“Spinobulbar muscular” NEXT atroph*) OR (“bulbo‐spinal” NEXT atroph*) OR MND):ti,ab,kw	1175
2 ‐ Values, preferences & QoL indicators	[mh “Patient preference”] OR [mh “attitude to health”] OR [mh “attitude to death”] OR [mh “attitude of health personnel”] OR (([mh patients] OR [mh caregivers] OR [mh family]) AND [mh “decision making”]) OR ((Patient* OR famil* OR carer* OR caregiver* OR care‐giver* OR caretaker* OR care‐taker* OR clinician* OR spouse* OR parent* OR carrier* OR doctor* OR specialist* OR (“health‐care” NEXT worker*) OR (healthcare NEXT worker*) OR (“health‐care” NEXT professional*) OR (healthcare NEXT professional*) OR provider* OR plwMND OR pwMND OR “people living with”) NEAR/6 (values OR valued OR prefer* OR desir* OR priorit* OR expectation* OR perspective* OR perceive* OR percept* OR view* OR attitude* OR accept* OR knowledge OR decision* or decid* OR choice* OR satisf* OR sentiment* OR motiv* OR approach*)):ti,ab,kw OR ((“patient‐reported experience” NEXT measure*) OR PREM OR (“patient‐reported outcome” NEXT measure*) OR ALSAQ‐40 OR EQ‐5D OR “Short Form Health Survey” OR SF‐36 OR “Health Utilities Index” OR HUI OR “Quality of Wellbeing” OR QWB):ti,ab,kw	192355
	1 AND 2	112

Clinical Trials (www.clinicaltrials.gov) (26/02/2025)	
1 ‐ MND	(“Gehrig* disease” OR “motor neuron* disease*” OR “progressive bulbar pals*” OR “progressive muscular atroph*” OR “spinal muscular atroph*” OR “hereditary spastic parapares*” OR “spinal and bulbar muscular atroph*” OR “Kennedy* disease*” OR “Kennedy* syndrome*” OR “bulbo‐spinal atroph*” or “flail arm ALS” OR “flail leg ALS” or “flail arm syndrome” OR “anterior horn cell disease*” OR “charcot disease*” OR “guam disease*” OR “bulbar pals*” OR “fazio‐londe* disease*” OR “fazio‐londe* syndrome*” OR “spinal amyotroph*” OR “spinal muscular atroph*” OR “bulbospinal neuronopath*” OR “hereditary motor neuronopath*” OR “infantile muscular atroph*” OR “juvenile muscular atroph*” OR “infantile acute form SMA” OR “werdnig hoffman* disease*” OR “motoneuron disease*” OR “motor system disease*” OR “ALS Dementia” OR “chinese paralytic syndrome*” OR “Aran Duchenne” OR “Duchennen Aran” OR “hereditary spastic paraplegia*” OR “hereditary autosomal dominant spastic paraplegia*” OR “hereditary autosomal recessive spastic paraplegia*” OR “hereditary motor and sensory neuropath*” OR “hereditary x linked recessive spastic paraplegia*“ OR “hypertrophic motor sensory neuropathy spastic paraplegia*” OR “spastic paraplegia type 2” OR “Charcot Marie” OR “Charcot Hoffman” OR “hereditary motor sensory neuropath*” OR “Dejerine sottas” OR “familial spastic para*“ OR “hereditary motor and sensory neuropath*” OR “hereditary sensorimotor neuropath*” OR “hereditary sensory and motor neuropath*” OR “neurogenic amyotroph*” OR “neurogenic atroph*” OR “neurogenic muscle atroph*” OR “neurogenic muscle syndrome*” OR “peroneal atroph*” OR “perone* muscle atroph*” OR “perone* muscular atroph*” OR “spastic hereditary paraglegia” OR “Struemell* disease*” OR “Struempell Lorrain” OR “Strumpel* disease*” OR “spinal amyotroph*” OR “bulbospinal neuronopath*” OR “distal spinal muscular atroph*” OR “hereditary motor neuronaopath*” OR “myelopathic muscular atroph*” OR “Spinobulbar muscular atroph*” OR “bulbo‐spinal atroph*“ OR MND OR “lateral scleros*”)
	AND
2 ‐ Values, preferences, & utility measures	(((patient* OR famil* OR carer* OR caregiver* OR “care‐giver*” OR caretaker* OR care‐taker* OR clinician* OR spouse* OR parent* OR carrier* OR doctor* OR specialist* OR “health‐care worker*“ OR “healthcare worker*” OR “health‐care professional*” OR “healthcare professional*” OR provider*) AND (value* prefer* OR desir* OR priorit* OR expectation* OR perspective* OR perceive* OR percept* OR view* OR attitude* OR accept* OR knowledge OR decision* or decid* OR choice* OR satisf* OR sentiment* OR motiv* OR approach*)) OR (“patient‐reported experience measure*” OR PREM OR “patient‐reported outcome measure*“ OR “ALSAQ‐40” OR “EQ‐5D” OR “Short Form Health Survey” OR “SF‐36” OR “Health Utilities Index” OR HUI OR “Quality of Wellbeing” OR QWB))
	= 500

WHO ICTRP (www.who.int.ictrp) (26/02/2025)	
1 ‐ MND	(“Gehrig* disease” OR “motor neuron* disease*“ OR “progressive bulbar pals*” OR “progressive muscular atroph*” OR “spinal muscular atroph*” OR “hereditary spastic parapares*” OR “spinal and bulbar muscular atroph*” OR “Kennedy* disease*” OR “Kennedy* syndrome*” OR “bulbo‐spinal atroph*“ or “flail arm ALS” OR “flail leg ALS” or “flail arm syndrome” OR “anterior horn cell disease*” OR “charcot disease*” OR “guam disease*” OR “bulbar pals*” OR “fazio‐londe* disease*” OR “fazio‐londe* syndrome*” OR “spinal amyotroph*” OR “spinal muscular atroph*” OR “bulbospinal neuronopath*” OR “hereditary motor neuronopath*” OR “infantile muscular atroph*” OR “juvenile muscular atroph*” OR “infantile acute form SMA” OR “werdnig hoffman* disease*” OR “motoneuron disease*” OR “motor system disease*” OR “ALS Dementia” OR “chinese paralytic syndrome*” OR “Aran Duchenne” OR “Duchennen Aran” OR “hereditary spastic paraplegia*” OR “hereditary autosomal dominant spastic paraplegia*” OR “hereditary autosomal recessive spastic paraplegia*” OR “hereditary motor and sensory neuropath*” OR “hereditary x linked recessive spastic paraplegia*” OR “hypertrophic motor sensory neuropathy spastic paraplegia*” OR “spastic paraplegia type 2” OR “Charcot Marie” OR “Charcot Hoffman” OR “hereditary motor sensory neuropath*” OR “Dejerine sottas” OR “familial spastic para*” OR “hereditary motor and sensory neuropath*” OR “hereditary sensorimotor neuropath*” OR “hereditary sensory and motor neuropath*” OR “neurogenic amyotroph*” OR “neurogenic atroph*” OR “neurogenic muscle atroph*” OR “neurogenic muscle syndrome*” OR “peroneal atroph*” OR “perone* muscle atroph*” OR “perone* muscular atroph*” OR “spastic hereditary paraglegia” OR “Struemell* disease*” OR “Struempell Lorrain” OR “Strumpel* disease*” OR “spinal amyotroph*” OR “bulbospinal neuronopath*” OR “distal spinal muscular atroph*” OR “hereditary motor neuronaopath*” OR “myelopathic muscular atroph*” OR “Spinobulbar muscular atroph*” OR “bulbo‐spinal atroph*” OR MND OR “lateral scleros*”)
	AND
2 ‐ Values, preferences, & utility measures	(((patient* OR famil* OR carer* OR caregiver* OR “care‐giver*” OR caretaker* OR care‐taker* OR clinician* OR spouse* OR parent* OR carrier* OR doctor* OR specialist* OR “health‐care worker*“ OR “healthcare worker*” OR “health‐care professional*” OR “healthcare professional*” OR provider*) AND (value* prefer* OR desir* OR priorit* OR expectation* OR perspective* OR perceive* OR percept* OR view* OR attitude* OR accept* OR knowledge OR decision* or decid* OR choice* OR satisf* OR sentiment* OR motiv* OR approach*)) OR (“patient‐reported experience measure*” OR PREM OR “patient‐reported outcome measure*“ OR “ALSAQ‐40” OR “EQ‐5D” OR “Short Form Health Survey” OR “SF‐36” OR “Health Utilities Index” OR HUI OR “Quality of Wellbeing” OR QWB))
	= 43 results

## Appendix III

Table A3

**Table A3:** Quantitative data extraction instrument.

**Design**	
Study ID	[Lead author last name, year of publication]
Other reports of this study identified?	[If multiple reports of this study were returned from the search strategy, include details of these reports here, including author, year, title, DOI etc.—This includes protocols of studies identified]
Time period study was conducted	[Dates provided by authors as to when the study was conducted]
Design	[PROM and QoL study] [Direct utility and health state value study] [Direct choice study] [Other quantitative study on outcome importance]
Health and QoL‐related outcomes assessed	[List all outcomes assessed that are relevant to this review]
**Setting**	
Country	
Sites/s	[Town, Region etc.]
Study locale	[In hospital‐care, outpatient care etc.] [As reported by the author]
Disease investigated	[If specified, what type or subtype of MND was investigated]
**Particpants**	
Category of participants under investigation	[Adults diagnosed with MND] [Caregivers and family members] [Clinicians] [Asymptomatic carriers]
Eligibility criteria of participants	[Who were eligible and why?]
Sampling strategy	[e.g., if random or consecutive, how? if through databases or physician enrolment, these details should be captured here].
Number of participants included	
Characteristics/demographics of participants included	[Transcribe as much details as possible, typically from characteristics of included studies table]
Response rate	
**Outcomes (this section may repeat if multiple outcomes assessed)**	
Instrument or method	[As reported by authors, e.g., forced choice, EQ‐5D (if quantitative), interview, focus group (if qualitative)].
Preference set used in translation of raw results	[As reported by authors, we will not be extracting raw results, only those that have been translated using a common preference set].
Reported format	[As reported by authors, e.g., Mean (SD or SE), Choice or proportion of choice, OR (95% CI)]
Result	[Report both numeric and narrative descriptions of the result can be reported here]

## Appendix IV

Table A4

**Table A4:** Qualitative data extraction instrument.

Design	
Study ID	[Lead author last name, year of publication]
Other reports of this study identified?	[If multiple reports of this study were returned from the search strategy, include details of these reports here, including author, year, title, DOI etc.—This includes protocols of studies identified]
Time period study was conducted	[Dates provided by authors as to when the study was conducted]
Design	[Qualitative study on outcome importance]
Qualitative methodology followed	[As stared by author, if unstated, put NR] [*note, specific methods are below per outcome]
Health and QoL‐related outcomes assessed	[List all outcomes assessed that are relevant to this review]
Setting	
Country	
Sites/s	[Town, Region etc.]
Study locale	[In hospital‐care, outpatient care etc.] [As reported by the author]
Disease investigated	[If specified, what type or subtype of MND was investigated]
Particpants	
Category of participants under investigation	[Adults diagnosed with MND] [Caregivers and family members] [Clinicians] [Asymptomatic carriers]
Eligibility criteria of participants	[Who were eligible and why?]
Sampling strategy	[e.g. if random or consecutive, how? if through databases or physician enrolment, these details should be captured here].
Number of participants included	
Characteristics/demographics of participants included	[Transcribe as much details as possible, typically from characteristics of included studies table]
Outcomes (this section may repeat if multiple outcomes assessed)	
Instrument or method	[As reported by authors, e.g. forced choice, EQ‐5D (if quantitative), interview, focus group (if qualitative)].
Findings	[The interpretation, conclusion or themes from the analysis of the qualitative data. These are supported by illustrations (below)].
Illustration	[Verbatim text from the included study of participant quotes]
Level of credibility	[Unequivocal] [Credible] [Unsupported]

## Appendix V

Table A5

**Table A5:** Data dictionary.

Term	Definition
**MND**	
Motor Neurone Disease (MND)	A group of progressive neurological disorders that destroy motor neurones, the cells that control skeletal muscle activity such as walking, breathing, speaking, and swallowing [43].
Amyotrophic Lateral Sclerosis (ALS)	Formerly known as Lou Gehrig's disease, is a neurological disorder that affects motor neurones, the nerve cells in the brain and spinal cord that control voluntary muscle movement and breathing [43].
Progressive Muscular Atrophy (PMA)	An uncommon subtype of ALS marked by slow but progressive damage to the lower motor neurones. It affects men more often than women, and usually symptoms begin later in life than typical ALS. People with PMA usually notice weakness in their hands or feet followed by spreads into other body regions. They may have weakness in the torso muscles and may have trouble breathing.
Progressive Bulbar Palsy (PBP)	Also known as progressive bulbar atrophy, is due to injury of the upper motor neurones in the brainstem or the lower motor neurones connected to the brainstem. The brainstem controls the muscles needed for swallowing, speaking, chewing, and other functions. PBP symptoms worsen over time and include trouble chewing, speaking, and swallowing. People with progressive bulbar palsy may also have weakness in the tongue and facial muscles, twitches, and a reduced gag reflex [43].
Primary Lateral Sclerosis (PLS)	A rare motor neurone disease characterized by the progressive degeneration of upper motor neurons, leading to muscle stiffness and weakness, particularly in the legs, arms, and tongue. Unlike other motor neurone diseases, PLS progresses more slowly and primarily affects upper motor neurones without involving lower motor neurones.
Hereditary Spastic Paraplegia (HSP)	Also known as familial spastic paraparesis, refers to a group of inherited disorders that involves weakness and spasticity, which is stiffness of the legs. These symptoms get worse over time. Early in the disease, there may be mild trouble walking and stiffness. These symptoms typically get worse slowly until a cane, walker, or wheelchair is needed [44].
MND with Frontal Temporal Dementia (MND/FTD)	Motor Neurone Disease with Frontotemporal Dementia (MND/FTD) is a condition where motor neurone disease coexists with frontotemporal dementia, affecting both movement and cognitive functions. This overlap leads to symptoms such as muscle weakness and atrophy, along with changes in personality, behaviour, and language.
Kennedy's Disease/Spinal and Bulbar Muscular Atrophy (SBMA)	A genetic disorder caused by mutations in the androgen receptor gene, leading to progressive muscle weakness and wasting in the limb and bulbar muscles. Symptoms often include tremors, muscle cramps, speech and swallowing difficulties, and signs of androgen insensitivity such as gynecomastia and testicular atrophy.
Monomelic Amyotrophy (MMA)	A rare motor neurone disease that primarily affects young adults, characterized by progressive muscle weakness and atrophy confined to one limb, usually an upper extremity. Unlike other motor neurone diseases, MMA typically has a benign course and does not progress to involve other limbs or lead to generalized disability.
**Eligible Population**	
Those diagnosed with MND or it's subtypes	Adults (18+) diagnosed with any type of MND, such as amyotrophic lateral sclerosis (ALS), progressive bulbar palsy (PBP), progressive muscular atrophy (PMA), primary lateral sclerosis (PLS), hereditary spastic paraparesis (HSP), MND with frontal temporal dementia (MND/FTD), Kennedy's disease/spinal and bulbar muscular atrophy (SBMA) and Hirayama disease/monomelic amyotrophy (MMA).
People with lived experience of MND	Caregivers and family members directly involved in care decisions. There will be no restrictions placed on the mode of caregiving (e.g. live‐in caregiver, full time caregiver, paid vs. unpaid etc.).
Clinicians	Those involved in the direct provision of treatment and care to people diagnosed with MND, or its subtypes as listed above (e.g. palliative care physicians to manage quality of life and end‐of‐life care or pulmonologists involved in the management of breathing difficulties and noninvasive ventilation).
Asymptomatic carriers	Also known as genetic carriers of MND. Defined as individuals that carry a mutation in a gene associated with MND, but do not exhibit symptoms of the disease themselves.
**Eligible Studies for Inclusion**	
Patient‐reported outcome measures (PROMs) and quality of life studies	Studies that quantitatively measure the lived experience of the population(s) of interest, in one or multiple domains that are directly relevant to patient satisfaction or quality of life. This may include studies recording PROMs, including those measuring quality of life. Quality of life measuring tools may be disease/topic specific questionnaires (such as the ALSAQ‐40 for MND28), or generic multi‐attribute utility instruments (MAUIs) such a EuroQol‐5 (EQ‐5D)29, Short Form‐36 Health Survey (SF‐36), Health Utilities Index (HUI) or Quality of Wellbeing (QWB). Where quality of life data has been captured using a validated tool and subsequently translated to a utility estimate (described below) using an external mapping or preference algorithm, both the raw and translated measures may be relevant.
Direct utility and health state value studies	Studies that directly measure how the population of interest perceive the value or quality of different health states by use of assigned numerical values (utility values) that reflect the desirability of living in those states. Utility values are typically on a scale where 1 represents perfect health, and 0 represent a state equivalent to death. In some instances, negative values can be used to indicate a state that is considered worse than death. Study methodologies that elicit direct measurement or valuation include survey techniques such as the Standard Gamble (SG), Time Trade‐Off (TTO) or Visual Analogue Scale (VAS). Although representing the same concept and nominally the same, utility values estimated by different methods of direct elicitation are poorly comparable to one another and to the indirect estimation of utility captured through MAUIs.
Direct choice studies	Studies that quantitatively examine the population of interest's choice, after presenting them with a description of hypothetical states or during decision making for their own actual health states.17 Studies may utilise a willingness to pay approach or conduct a randomised controlled trial of preferences (whereby there is a randomised group [participants randomised to treatment groups as per normal] and a preferences group [participants with strong preference for a treatment are allocated to their preferred treatment]). They may also utilise a direct/forced choice exercise where the population of interest is prompted to make a choice from a set of options or rank a set of alternatives to enable best‐worst scaling.
Other studies utilising quantitative methodology to assess outcome importance	Studies that may employ any other quantitative methodology (e.g., bespoke questionnaires or other nonutility measurement techniques) whereby the patients values and preferences regarding their health‐related outcomes are examined.
Qualitative studies on outcome importance	Qualitative research studies that have explored the population of interest's values and preferences related to different health‐related outcomes. However, qualitative research that explores the population of interest's values and preferences related to treatment options/modalities or provision of care were not eligible for inclusion.
Studies utilised mixed methodology	Studies will also be eligible for inclusion where they have employed a range of eligible methodologies, (e.g., mixed‐methods studies that include both quantitative and qualitative data collection methods would be eligible, if the methodologies followed are consistent with those listed above).
